# A National Cancer Grid pooled procurement initiative, India 

**DOI:** 10.2471/BLT.23.289714

**Published:** 2023-06-29

**Authors:** C S Pramesh, Manju Sengar, Sumedha Patankar, Girish Chinnaswamy, Sudeep Gupta, M Vijayakumar, Sanjeev Sood, Anil N Sathe, Venkatraman Radhakrishnan, Prasanth Ganesan, Krishna Mohan Mallavarapu, Rajendra A Badwe

**Affiliations:** a Tata Memorial Hospital, Tata Memorial Centre, affiliated with the Homi Bhabha National Institute, Dr Ernest Borges Road, Parel, Mumbai 400012, India.; b Tata Memorial Centre, affiliated with the Homi Bhabha National Institute, Mumbai, India.; c Zulekha Yenepoya Institute of Oncology, Yenepoya (Deemed to be university), Mangalore, India.; d Cancer Institute (Women in India) Chennai, India.; e Jawaharlal Institute of Postgraduate Medical Education and Research, Puducherry, India.; f Basavatarakam Indo American Cancer Hospital & Research Institute, Hyderabad, India.

## Abstract

In health systems with little public funding and decentralized procurement processes, the pricing and quality of anti-cancer medicines directly affects access to effective anti-cancer therapy. Factors such as differential pricing, volume-dependent negotiation and reliance on low-priced generics without any evaluation of their quality can lead to supply and demand lags, high out-of-pocket expenditures for patients and poor treatment outcomes. While pooled procurement of medicines can help address some of these challenges, monitoring of the procurement process requires considerable administrative investment. Group negotiation to fix prices, issuing of uniform contracts with standardized terms and conditions, and procurement by individual hospitals also reduce costs and improve quality without significant investment. The National Cancer Grid, a network of more than 250 cancer centres in India, piloted pooled procurement to improve negotiability of high-value oncology and supportive care medicines. A total of 40 drugs were included in this pilot. The pooled demand for the drugs from 23 centres was equivalent to 15.6 billion Indian rupees (197 million United States dollars (US$)) based on maximum retail prices. The process included technical and financial evaluation followed by contracts between individual centres and the selected vendors. Savings of 13.2 billion Indian Rupees (US$ 166.7million) were made compared to the maximum retail prices. The savings ranged from 23% to 99% (median: 82%) and were more with generics than innovator and newly patented medicines. This study reveals the advantages of group negotiation in pooled procurement for high-value medicines, an approach that can be applied to other health systems.

## Résumé

Lorsque les systèmes de santé reçoivent peu de fonds publics et que leurs processus d'achat sont décentralisés, le prix et la qualité des médicaments contre le cancer ont un impact direct sur l'accès aux traitements efficaces contre la maladie. Des facteurs tels que l'application de prix différenciés, les négociations en fonction des volumes ainsi que la confiance placée dans des génériques bon marché dont la qualité n'a pas été évaluée peuvent entraîner des décalages entre l'offre et la demande, d'énormes dépenses non remboursables pour les patients et de piètres résultats thérapeutiques. Bien que les acquisitions groupées de médicaments puissent contribuer à résoudre certains de ces problèmes, le suivi du processus d'achat requiert un engagement considérable au niveau administratif. Les négociations collectives en vue de fixer les tarifs, l'établissement de contrats types assortis de conditions générales standardisées, mais aussi les achats effectués par des hôpitaux en particulier peuvent également faire baisser les coûts et améliorer la qualité sans nécessiter d'importants investissements. Le National Cancer Grid, un réseau réunissant plus de 250 centres d'oncologie en Inde, a testé un dispositif d'achat groupé visant à assurer une meilleure négociabilité pour des médicaments et soins de soutien essentiels contre le cancer. Au total, 40 substances ont été prises en compte dans ce projet pilote. La demande groupée en médicaments émise par 23 centres équivalait à 15,6 milliards de roupies indiennes (197 millions de dollars américains) d'après le prix maximal de vente au détail. Ce processus prévoyait une évaluation technique et financière, puis des contrats entre chaque centre et les distributeurs sélectionnés. Des économies de 13,2 milliards de roupies indiennes (166,7 millions de dollars américains) ont pu être réalisées par rapport au prix maximal de vente au détail. Ces économies étaient comprises entre 23 et 99% (médiane: 82%) et concernaient davantage les médicaments génériques que les marques et les médicaments récemment brevetés. La présente étude révèle les avantages que représentent les négociations collectives lors des achats groupés de médicaments essentiels, une approche applicable à d'autres systèmes de santé.

## Resumen

En los sistemas sanitarios con escasa financiación pública y procesos de adquisición descentralizados, el sistema de fijación de precios y la calidad de los medicamentos contra el cáncer afectan directamente al acceso a una terapia eficaz contra dicha enfermedad. Factores como los diferentes sistemas de determinación de precios, la negociación en función del volumen y la dependencia de genéricos de bajo precio sin evaluación de su calidad pueden generar retrasos en la oferta y la demanda, elevados gastos para los pacientes y malos resultados en el tratamiento. Aunque la adquisición conjunta de medicamentos puede ayudar a abordar algunos de estos retos, el seguimiento del proceso de adquisición requiere una inversión administrativa considerable. La negociación colectiva a la hora de determinar los precios, la emisión de contratos unificados con términos y condiciones estandarizados y la adquisición por parte de algunos hospitales también reducen los costes y mejoran la calidad sin necesidad de realizar una gran inversión. La Red Nacional de Cáncer, una red que cuenta con más de 250 centros oncológicos en la India, puso a prueba la adquisición conjunta con el fin de mejorar la negociabilidad de medicamentos oncológicos y de tratamiento complementario que resultaban costosos. En esta prueba piloto se incluyó un total de 40 medicamentos. La demanda conjunta de medicamentos por parte de 23 centros fue equivalente a 15 600 millones de rupias indias (197 millones USD) según los precios minoristas máximos. El proceso incluyó una evaluación técnica y financiera, así como contratos entre centros independientes y proveedores seleccionados. Se logró un ahorro de 13 200 millones de rupias indias (166,7 millones USD) en comparación con los precios minoristas máximos. El ahorro osciló entre el 23 y el 99% (media: 82%) y fue más alto con los medicamentos genéricos que con los de marca y los recién patentados. Este estudio pone de manifiesto las ventajas de la negociación colectiva en lo que respecta a la adquisición conjunta de medicamentos costosos, un enfoque que se puede aplicar a otros sistemas sanitarios.

## ملخص

في النظم الصحية ذات التمويل العام المحدود وعمليات الشراء اللامركزية، يؤثر كل من التسعير وجودة الأدوية المضادة للسرطان، بشكل مباشر على الوصول إلى العلاج الفعال المضاد للسرطان. إن عوامل مثل التسعير التفاضلي، والتفاوض المعتمد على الحجم، والاعتماد على الأدوية المكافئة ذات الأسعار المنخفضة دون أي تقييم لجودتها، يمكن أن تؤدي إلى تأخيرات العرض والطلب، ونفقات شخصية للمرضى، ونتائج سيئة للعلاج. في حين أن الشراء المجمع للأدوية يمكن أن يساعد في مواجهة بعض هذه التحديات، فإن مراقبة عملية الشراء تتطلب استثمارًا إداريًا كبيرًا. كما أن التفاوض الجماعي لتحديد الأسعار، وإبرام عقود موحدة بشروط وأحكام قياسية، والشراء بواسطة المستشفيات الفردية، يقلل أيضًا من التكاليف ويُحسّن الجودة دون استثمارات كبيرة. قامت الشبكة الوطنية للسرطان، وهي شبكة تضم أكثر من 250 مركزًا للسرطان في الهند، بإطلاق تجربة الشراء الجماعي لتحسين إمكانية التفاوض على أدوية الأورام عالية القيمة، وأدوية الرعاية الداعمة. تم تضمين إجمالي 40 دواءً في هذه التجربة الأولية. كان الطلب المجمع على الأدوية من 23 مركزًا هو ما يعادل 15.6 مليار روبية هندية (197 مليون دولار أمريكي) بناءً على أقصى أسعار للتجزئة. وتضمنت العملية التقييم الفني والمالي، وبعده عقود بين المراكز الفردية والموردين المحددين. تم توفير 13.2 مليار روبية هندية (166.7 مليون دولار أمريكي) مقارنة بأقصى أسعار للتجزئة. تراوحت المبالغ المتوفرة من 23 إلى %99 (المتوسط: %82) وكانت مع الأدوية المكافئة أكثر من العلامة التجارية والأدوية الحاصلة على براءة اختراع حديثًا. تكشف هذه الدراسة عن مزايا التفاوض الجماعي في الشراء المجمع للأدوية عالية القيمة، وهو أسلوب يمكن تطبيقه على الأنظمة الصحية الأخرى.

## 摘要

在缺乏公共资金和采购流程比较分散的卫生系统中，抗癌药物的定价和质量会直接影响获得有效抗癌治疗的机会。差别定价、依赖数量的价格谈判和依赖低价通类药物而不对其质量进行任何评估等因素可能导致供需滞后、患者自付费用高以及治疗效果差。虽然集中采购药品有助于解决其中一些此类挑战，但监测采购过程需要大量的行政投资。通过集体谈判确定价格，签发具有标准化条款和条件的统一合同，然后由医院进行单独的采购，可以在不进行重大投资的情况下降低成本并提高质量。印度国家癌症网格是一个由 250 多个癌症中心组成的网络，该网格试点运行了集中采购计划，以改善高价值肿瘤药物和支持性护理药物的价格可协商性。此次试点项目共纳入了 40 种药物。根据最高零售价格计算，23 个中心的药物总需求量相当于 156 亿印度卢比（1.97 亿美元）。该过程包括进行技术评估和财务评价，然后由不同中心与选定供应商单独签订合同。与最高零售价格相比，共节省了 132 亿印度卢比（1.667 亿美元）。节省幅度为 23% 到 99% 不等（中值：82%），并且通类药物的节省幅度大于品牌药物和新专利药品。本研究揭示了在高价值药物的集中采购中进行小组谈判具有一定的优势，这种方法可以应用于其他卫生系统。

## Резюме

В системах здравоохранения с небольшим финансированием из государственного бюджета и децентрализованными процессами закупок цена и качество противораковых препаратов напрямую влияют на доступ к эффективной противоопухолевой терапии. Такие факторы, как дифференцированное ценообразование, переговоры в зависимости от объема и использование недорогих непатентованных препаратов без оценки их качества, могут привести к разрыву между спросом и предложением, высоким расходам из собственных средств пациентов и ухудшению результатов лечения. В то время как совместные закупки лекарственных средств могут помочь решить некоторые из этих проблем, мониторинг процесса закупок требует значительных административных расходов. Кроме того, снижение затрат и повышение качества без значительных инвестиций достигается за счет групповых переговоров по установлению цен, заключения единых контрактов со стандартизированными условиями и закупок отдельными больницами. Национальная сеть по борьбе с раком, объединяющая более 250 онкологических центров в Индии, провела экспериментальную закупку для повышения доступности дорогостоящих лекарственных препаратов для лечения онкологических заболеваний и оказания сопроводительной терапии. В общей сложности в эксперимент было включено 40 препаратов. Исходя из максимальных розничных цен, совокупный спрос на лекарственные препараты в 23 центрах составил 15,6 млрд индийских рупий (197 млн долларов США). Этот процесс предусматривал техническую и финансовую оценку с последующим заключением контрактов между отдельными центрами и выбранными поставщиками. По сравнению с максимальными розничными ценами была достигнута экономия в размере 13,2 млрд индийских рупий (166,7 млн долларов США). Экономия составляла от 23 до 99% (медиана: 82%) и была более существенной при использовании непатентованных препаратов по сравнению с препаратами с торговым названием и новыми запатентованными препаратами. В данном исследовании раскрываются преимущества групповых переговоров при совместных закупках дорогостоящих лекарственных средств, причем такой подход может быть применен и в других системах здравоохранения.

## Introduction

Systemic anti-cancer therapy constitutes a key component of cancer management.^1^ Over the years, advances in our understanding of cancer biology, coupled with discovery of different classes of anti-cancer agents, the use of more appropriate combinations of anti-cancer drugs, and rationalized sequencing of treatment, has led to gains in survival.^1^ Ensuring access and affordability of these drugs is the key factor in translating the survival gains observed in clinical trials to real-world scenarios.^2^^,^^3^

Every two years, the World Health Organization (WHO) releases an updated essential medicines list for evidence-based cancer treatment.^4^^–^^7^ The purpose of the essential medicines list is to guide individual national formularies and to facilitate universal access to these drugs. However, one of the major obstacles to the implementation of this guidance in India is the unaffordable prices of many anti-cancer drugs.^8^

Universal access to essential cancer medicines is limited in many low- and middle-income countries.^9^ For example, Indian oncologists from both private and public hospitals reported substantial out-of-pocket-expenditure for even conventional cytotoxic drugs, and catastrophic expenditure for drugs like rituximab and trastuzumab.^10^ Some of the major reasons cited are: unregulated prices and procurement systems in private hospitals; poor- quality drugs obtained on the basis of lowest pricing; and frequent stock-outs due to supply chain issues. 

Spending on systemic anti-cancer therapies constitutes a major proportion of health-care expenditure in cancer treatment.^11^ Escalating drug costs are increasingly recognized as hurdles to effective treatments, even in high-income countries with universal free health care or insurance-based reimbursement and co-payment models.^11^^,^^12^ In national health systems that rely on co-payments or limited public funding, drug pricing contributes considerably to out-of-pocket expenditure for patients.^13^ These drug pricing models are a concern in low- and middle-income countries where there is the dual problem of a major cancer burden and limited health budgets.^2^

In the market, a phenomenon exists where innovators set high prices for their products, targeting affluent customers who are less sensitive to price changes and prioritize quality. In contrast, branded generics provide lower-priced alternatives with somewhat uncertain quality, catering to customers with lower incomes who are more price-sensitive.^14^ Several approaches can help in developing fair pricing mechanisms for anti-cancer drugs including cost-based pricing, value-based pricing, reference pricing and pricing based on tendering and negotiations.^15^ To achieve this, some countries have adopted an approach of managed entry agreements or have set maximum allowable prices for medicines.^16^

Drug procurement in public and private hospitals often happens at the individual hospital level or, at best, at the level of a public organization. The primary criterion for selection in these cases is often the lowest price available for the drug. As a result of suboptimal purchasing processes and limited negotiation capacity with the pharmaceutical industry, the current approach to drug procurement can lead to unfavourable commercial terms for low-volume centres, drug shortages, and the use of poor-quality generics.^17^ A primary result of these unfavourable terms for drug pricing is excessive spending at the individual, regional and national levels.^18^^,^^19^

Given these considerations, optimizing the drug procurement processes is important to ensure a regular supply of high-quality anti-cancer and supportive care drugs at affordable prices. In Europe, numerous pilot programmes and evaluations for multicountry pooled procurement for medicines failed to yield positive results.^20^^,^^21^ Some of the challenges faced were legislative and organizational – such as differences in health-care systems between participating countries.^20^^,^^21^

The Centre for Global Development evaluated the effect of centralized procurement on drug prices, using data from seven low- and middle-income countries with diverse drug procurement systems. The study indicated that centralized procurement could result in considerable lowering of drug prices, including several anti-cancer drugs.^22^^,^^23^ However, centralized procurement systems require considerable administrative and managerial resources. A pooled procurement approach that is less resource-intensive and sustainable without significant investment is the WHO-suggested group contracting approach.^24^ This approach involves collective negotiation of drug prices and selection of suppliers and distributors, although the actual purchasing is done by the individual member organizations.

Here, we describe how we piloted a group negotiation approach for high-value anti-cancer and supportive care drugs. Centres included belong to the National Cancer Grid of India, a large network of over 250 cancer centres, research organizations and patient groups that deliver uniform, high-quality and affordable care to all patients with cancer.^25^ The member centres care for almost two thirds of all patients with cancer in India. These volumes place the network in a unique position to use pooled procurement and group negotiation to ensure uninterrupted supply of high-quality drugs at affordable prices to member centres.

## Negotiation approach

**Fig. 1:**
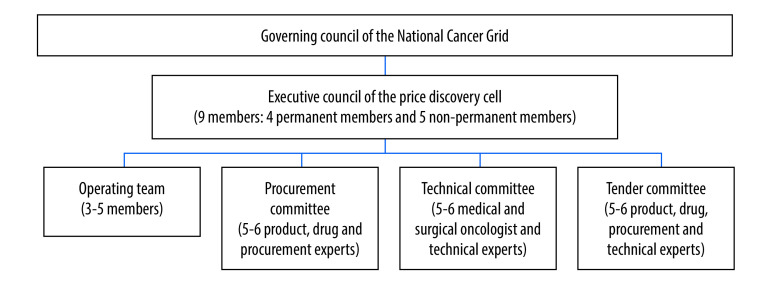
Governance structure of the price discovery cell, National Cancer Grid, India

In September 2019, the network initiated a price discovery cell to help aggregate demands of its members and negotiate for lower overall procurement costs of quality drugs, equipment, medical journals and other requirements. The governing structure for the price discovery cell is presented in Fig. 1. 

To determine the drugs that had the greatest impact on the annual procurement budget, we conducted a pilot study at three prominent cancer centres in India. This study considered both the cost and volume of individual drug molecules. We further refined the list in discussion with a group of oncologists. Following that, we circulated a letter of intent containing the list of chosen drugs to the leadership of the network centres. The purpose was to gauge their interest in participating in the pooled procurement, and to gather their input on any additional drugs they deemed necessary to be included in the list. For each of the selected drugs, we identified common dosage and stocking practices to ensure completeness of the list and to avoid difficulties in dosing or wastage after prescription. Once the list of drugs and participating centres was finalized, we collected data on demand for each listed drug in stock-keeping units. We based projected volumes on their usage in the previous year and predicted future changes in use. To document the demand, we designed a template that separately captured the requirements for both innovator and generic versions of each stock-keeping unit.

To facilitate the process of determining fair prices, we established the concept of reserve price. The reserve price is the maximum price at or below which the participating members of the network would agree to purchase the drug through group negotiations. If the final price determined by the price discovery cell exceeded the reserve price in this negotiation cycle, the members could opt out of the procurement process. 

Each participating centre was requested to provide the cost of procurement along with details of the brand, generic and molecules from the selected stock-keeping units for the previous year. Based on this data, the reserve price was benchmarked using the lowest price for each listed drug. Different reserve prices were set for generics and innovator molecules. The reserve prices were then circulated to the participating centres for their agreement to procure. All participating centres agreed to a minimum purchase commitment. This commitment ensured that the demand committed by the centre in the demand collation template is a firm committed volume, which the price discovery cell could indicate to pharmaceutical companies as likely annual volumes.

In addition, the centres signed a non-disclosure agreement to safeguard the information exchanged during the discovery process and initiation of reserve prices. We created a request for proposal with details of the tendering process, steps involved, required documents and terms and conditions. An online tendering and evaluation platform (Nextenders (India) Private limited, Mumbai, India) was used for the submission of tender forms and subsequent evaluations of those tenders. 

We evaluated the submitted tenders in three stages: (i) prequalification; (ii) technical evaluation; and (iii) financial evaluation (Box 1). Technical evaluation required clearance at the prequalification stage; similarly, financial bids were opened only where the company and drugs qualified the technical evaluation. The criteria for prequalification included financial turnover; good manufacturing practices compliance; ability to deliver to different geographical locations; and the performance of the company including any reports of non-compliance in the past. The criteria for technical evaluation included parameters to assess the processes; transparency; market standing; research commitment; compliance with regulations; cold chain maintenance; and other surrogates for drug quality and company standards. The financial evaluation was based on the price quoted per unit of the drug, applicable taxes, and whether the drug was listed under drug pricing control by the National Pharmaceutical Pricing Authority.^26^ The tender committee developed and reviewed financial comparative statements.

Box 1 **Evaluation stages in the pooled procurement of cancer medicine, India, 2019–2020**

**1. Pre-qualification questions, to be filled out by bidder:**

(i) name of the bidder; (ii) provide average annual turnover of the firm over the past 3 years (in Indian rupees); (iii) attach certificate of annual turnover authenticated by chartered accountant for the last 3 financial years (2018–2019, 2017–2018 and 2016–2017); (iv) attach balance sheet and profit and loss account of the firm for the last 3 financial years (2018–2019, 2017–2018, 2016–2017); (v) if available, attach the latest copy of a valid WHO Good Manufacturing Practices certificate; (vi) attach latest no conviction certificate by United States FDA; (vii) attach performance certificate for past 3 years issued by FDA of any relevant state; (viii) provide name and address of all board partners and director(s) of the firm; (ix) enter permanent account number, and attach copy of permanent account number registration; (x) enter goods and service tax number, and attach copy of goods and service tax registration; (xi) attach latest income tax assessment certificate (preferably for financial year 2018–2019); (xii) attach the proof of Factories Act registration, or shops and establishments registration, or small-scale industries registration, or micro, small and medium enterprises registration, as applicable; (xiii) attach an affidavit by the firm that the firm has not been debarred or blacklisted by any general or private hospital; (xiv) attach national electronic fund transfer form duly signed by bank authority for earnest money deposit refund; (xv) attach earnest money deposit receipt (scanned copy of demand draft or national electronic fund transfer receipt, whichever is applicable); (xvi) confirm willingness to retain the contract rates for one more year after the rate contract period is over; and (xvii) confirm capability to deliver to all of the given locations.

**2. Technical qualification parameters for vendors:**

(i) Number of innovator drugs already approved and marketed in India, Europe, United States and/or other countries (may include non-oncology drugs); (ii) company’s total annual research budget (in Indian rupees); (iii) percentage of annual turnover of company's annual budget spent on research; (iv) does the company have a separate medical department? (v) does the company have a United States FDA or European Medical Agency inspection and approved manufacturing facility in India (should be wholly owned and operated by the company)? If yes, provide name and location; (vi) does the company have a fully owned and operated manufacturing facility in the United States or Europe? If yes, provide name and location; (vii) number of years since marketing approval in India for this brand, provide initial year of launch; (viii) does the company manufacture the drug in a fully owned and operated facility? If yes, then name and location; (ix) details on cold chain supply logistics; (x) type of manufacturing facility; (xi) manufacturing facility address; (xii) compliance of the active pharmaceutical ingredient with the United States pharmacopeia and/or the EU pharmacopeia; (xiii) provide standard operating procedures if they exist; (xiv) batch rejection rate (or all drugs manufactured at the plant in the year); (xv) having WHO Good Manufacturing Protocol Certificate; (xvi) having United States FDA certification for a wholly owned and operated manufacturing facility; (xvii) patent validity for original molecule; (xviii) a list of the number of chemotherapy drugs marketed by the company (not applicable for non-oncology molecules); (xix) is this the first approved Indian brand for this drug? (xx) submit document if this brand is marketed in the United States, Europe, Asia Pacific Region (excluding China) or Japan; (xxi) provide source of raw material for this brand, including name of company and country; (xxii) provide information if clinical data is available using this brand in any approved indication; (xxiii) provide reference for PubMed® publications, if any; (xxiv) unpublished data and regulatory evidence on file (provide copy); (xxv) provide a copy if the product is therapeutic, bio or chemical equivalent; and (xxvi) is this an original innovator brand?

**3. Financial evaluation parameters for vendors**

(i) name of the molecule; (ii) strength and dosage; (iii) formulation; (iv) if drug covered under drug price control; (v) base unit per mg cost; (vi) harmonized system of nomenclature code; (vii) applicable goods and service tax, %; (viii) pack size, number of base units per sellable pack; (ix) pre-tax price per pack, in Indian rupees; (x) free-of-cost units provided per pack purchased; (xi) applicable discount, %; (xii) customs, %; (xiii) excise tax, %; (xiv) any other applicable taxes, %; (xv) final price per pack, in Indian rupees; (xvi) final rate per base unit; and (xvii) maximum retail price, including all taxes.

EU: European Union; FDA: Food and Drug Administration; WHO: World Health Organization.

**Fig. 2:**
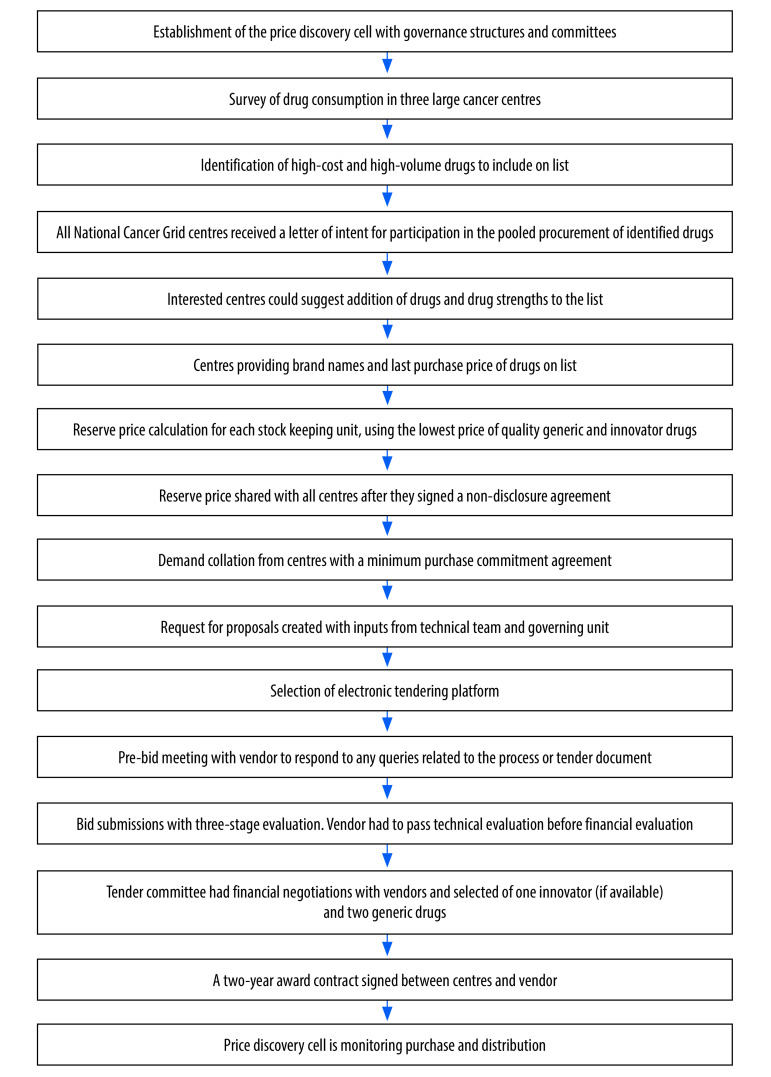
Flowchart of the price discovery cell process for pooled procurement of cancer medicine, India, 2019–2020

The price discovery cell reviewed all comparative pricing options, and ultimately two suppliers with the lowest-priced generic and innovator molecules were invited for commercial discussions with members of the price discovery cell. On the basis of commercial discussions, the final price along with terms and conditions were finalized with the selected vendor(s). On finalization of vendor(s), the price discovery cell facilitated the signing of an agreement for sale between each centre and vendor. The purpose of implementing an agreement for sale was to prevent vendors from breaching the terms and conditions established with the network. As part of the agreement, centres had to sign a purchase order with the vendors. The vendors committed to supplying enough drugs to all centres, regardless of their geographical location. The validity of the negotiated price was for two years. Fig. 2 provides the step-wise description of the entire pooled procurement.

## Outcome of negotiations

From September 2019 to October 2020, the price discovery cell conducted pooled procurement activities. A total of 40 drugs (chemotherapy, targeted therapy, supportive care, antibiotics and antifungal) and 85 stock-keeping units, were shortlisted for inclusion in consultation with participating centres (Table 1). During the technical evaluation, dasatinib was withdrawn from the selected list of drugs because the single source supplier never provided a quote, and no generics were available. Cetuximab (marketed by a single vendor with no generics) and iohexol (no quotations received) were also withdrawn. 

**Table 1:** Selected drugs for group negotiation under pooled procurement, India, 2019–2020

Drug, by class	Stock-keeping unit	Route of administration	Listed in national list of essential medicines
**Anticoagulant**			
Enoxaparin sodium	20, 40, 60 and 80 mg	Parenteral	Yes
**Antiemetic**			
Aprepitant	80 and 125 mg	Oral	No
**Antifungal**			
Posaconazole	200 mg	Syrup	No
Voriconazole	50 and 200 mg	Oral and parenteral	No
**Antimicrobial**			
Meropenem	500 and 1000 mg	Parenteral	Yes
Teicoplanin	200 and 400 mg	Parenteral	No
**Antineoplastic**			
Bevacizumab	100 and 400 mg	Intravenous	No
Bortezomib	1.0, 2.0, 2.5 and 3.5 mg	Parenteral	Yes
Capecitabine	500 mg	Oral	Yes
Carboplatin	150 and 450 mg	Intravenous	Yes
Cetuximab	100 mg	Intravenous	No
Cisplatin	10 and 50 mg	Intravenous	Yes
Crizotinib	200 and 250 mg	Oral	No
Cytarabine	100, 500 and 1000 mg	Intravenous	Yes
Dasatinib	50 and 70 mg	Oral	No
Docetaxel	20, 80 and 120 mg	Intravenous	Yes
Doxorubicin	10 and 50 mg and 50 mg (powder to be reconstituted)	Intravenous	Yes
Epirubicin	10, 50 and 100 mg	Intravenous	No
Erlotinib	100 and 150 mg	Oral	No
Gefitinib	250 mg	Oral	Yes
Gemcitabine	200, 1000 and 1400 mg	Intravenous	Yes
Ifosfamide	1000 and 2000 mg	Intravenous	Yes
Imatinib	100 and 400 mg	Oral	Yes
Imipenem+cilastatin	500 mg	Intravenous	No
Irinotecan	40 and 100 mg	Intravenous	Yes
Lapatinib	250 mg	Oral	No
L-Asparaginase	5000 and 10 000 IU	Intravenous	Yes
Nilotinib	150 and 200 mg	Oral	No
Oxaliplatin	50 and 100 mg	Intravenous	Yes
Paclitaxel	30, 100, 260 and 300 mg	Intravenous	Yes
Pemetrexed	100 and 500 mg	Intravenous	No
Rituximab	100 and 500 mg	Intravenous	Yes
Sunitinib	12.5, 25.0 and 50.0 mg	Oral	No
Temozolomide	20, 100 and 250 mg	Oral	Yes
Trastuzumab	150 and 440 mg	Intravenous	Yes
**Growth factor**			
Filgrastim	300 μg	Parenteral	Yes
**Hormonal agent**			
Leuprolide acetate	3.75, 11.25, 22.50 and 45.00 mg	Parenteral	Yes
**Intravenous contrast**			
Iohexol	300 mg	Intravenous	Yes
**Parenteral iron**			
Ferric carboxymaltose	100 and 500 mg	Intravenous	No

IU: international unit.

A total of 23 network centres participated in pooled procurement piloting, including both public and private centres. The participating centres were distributed across all the geographic regions of the country, and were a mix of small, mid-size and large-volume cancer centres. The main reasons for non-participation from other centres included pre-existing rate contracts; outsourcing of pharmacy to a private vendor; government scheme-specific funding for oncology drugs; and/or concerns about the clinical acceptance of procured brands by individual oncologists in cancer centres. For the participating centres, the pooled demand for the drugs was equivalent to 15.6 billion Indian rupees (US$ 197 million ) as per the maximum retail price and 1.6 billion Indian rupees (US$ 20 million) as per the reserve price.

We organized a pre-bid meeting with all potential vendors to clarify any questions they had regarding the tendering and evaluation, pooled procurement financing, and distribution processes. After debriefing, a total of 46 vendors submitted their bids for tendering on the electronic platform. After the pre-qualification stage, we selected 33 vendors for technical evaluation. From these bids, we selected 28 vendors for financial evaluation. The price discovery cell awarded 24 vendors a contract, but only 21 vendors agreed to supply the selected drugs at pooled procurement rates. The remaining three cited their inability to supply the drugs at pooled prices due to fluctuations in manufacturing costs such as raw materials and other administrative complications, including but not limited to the sale of marketing rights to other companies. 

We calculated the cost implications of pooled procurement based on the maximum retail price quoted by selected vendors and the reserve price set by the price discovery cell. A total of 13.2 billion Indian rupees (US$ 166.7 million) were saved compared to the maximum retail price; and 337 million Indian rupees (US$ 4.2 million) as per the reserve price. The savings ranged from 23% to 99% (median: 82%) on maximum retail price, with more savings observed among generics than innovator drugs. There was no observed difference based on the type of molecule. The entire process took a total of one year from the establishment of the price discovery cell to signing of the contracts. 

## Feasibility of approach

In this study, we demonstrate the feasibility of pooled procurement for anti-cancer and supportive therapies using group negotiation and concurrence on pricing. The price discovery cell achieved considerable savings, both on the reserve price and the maximum retail price for 40 high-value and high-volume drugs used in patients with cancer, including conventional cytotoxic drugs, targeted therapies, antibiotics, antifungals, anti-emetics and growth factors. 

The potential impact of cost savings is huge, in not only improving the affordability of care and decreasing out-of-pocket costs for patients, but allowing for the re-allocation of drug procurement funds towards other initiatives to deliver high-quality care. Savings were not restricted solely to generic drugs; there were also savings observed for innovator drugs, albeit to a lesser degree. These savings are notable because they were achieved without compromising on quality, due to strict standards imposed on both the drugs and the companies. 

A study analysing procurement systems in seven low- and middle-income countries showed at least 15% reduction in procurement prices on essential drugs when using public pooled procurement.^22^ However, our pilot project yielded greater savings. This outcome suggests that the concentration of demand significantly strengthened our negotiating power, while the centralized negotiation approach, combined with larger purchase quantities, allowed us to secure substantial price discounts.

In addition to cost savings at the centralized level, this approach also benefited individual patients across different regions of the country, demonstrating that substantial cost savings can be achieved even with varying geographic delivery regions and procurement volumes. We have shown that pooled procurement can enable access to high-quality drugs at a lower cost for patients in both public and private hospitals. This achievement needs to be interpreted against the backdrop of challenges to access and affordability of cancer medicines in India and other low- and middle-income countries. 

Initiatives like the price discovery cell have the potential, through a rigorous and credible system of pooled procurement through group consensus, to ensure high-quality, timely supply of essential drugs at affordable prices. Nearly all of the drugs included in the price discovery cell lists for negotiation were essential anti-cancer drugs (Table 1). 

Providing equal access to these drugs also has the potential to improve overall treatment outcomes. Reductions in drug pricing could also lower treatment abandonment rates, which are known to be associated with lower survival rates.^27^ If successful, the approach could also reduce the financial burden individual patients face through reduction of out-of-pocket expenditure, and increased public health spending via government-funded schemes. We therefore believe that the process and framework followed in pooled procurement by the network can not only help India reduce the cost of national oncology care, but that this approach can also be applied in other countries to bring down the cost of care.

Pooled procurement has been in practice in some European countries in the form of regional, national and multicountry procurement for decades.^20^^,^^21^ European programmes are largely limited to specific medicines or vaccines procured via the national health scheme, with few European countries procuring all of their drugs and supplies through pooled procurement. Based on the European evidence, the recommended process is to award the contract to the most economically advantageous tender; however this process, often taking place at regional or hospital level, ignored the quality of the drugs procured. In some procurement models we reviewed, the national level focused on ensuring availability of medicines and supply security rather than cost savings for patients and centres. Therefore, data on the economic impact of pooled procurement on patients, centres and countries are sparse. 

One limitation we observed in the European system is that suppliers of innovator drugs and managers of hospital-level formulary often act as a deterrent for the purchase of biosimilars.^28^ Several of these limitations can be addressed by following the approach of the price discovery cell, which includes high-value and essential medicines, and tenders from suppliers and vendors of both generic and innovator drugs. We based selection primarily on drug quality, whereas price was considered only for those which qualified the technical (quality) evaluation. By establishing and setting the reserve price before the tendering process, using the lowest-priced brand that met quality criteria among the participating centres, both the centres and oncologists gained confidence in the process.

## Challenges moving forward

Our experience highlights some of the inherent barriers to pooled procurement, including single vendor availability; scepticism from some centres; differences at organizational level within the centres; need for dedicated staff; determination of appropriate price; quality determinants; and monitoring of vendors throughout the contract to ensure adherence and supply across all the hospitals.

While the network consists of more than 250 cancer centres; only 23 centres participated in the first round of pooled procurement. Some of the reasons cited by members for not participating are administrative scepticism about the process and degree of price discounting that would be acquired, as well as concerns about the quality of drugs that would be procured as a result of these pooled negotiations. However, the success we obtained in the first round allayed the fears of administrators and oncologists in the non-participating centres regarding drug quality and pricing. For the upcoming second round, we have had a considerable response rate from the remaining centres to participate in our process.

We required one year to complete the pilot process due to administrative challenges both at the hospital and vendor level because of the novel coronavirus disease 2019 (COVID-19) pandemic. The quality parameters we used for shortlisting vendors and drugs were based on certain surrogates (Box 1). To strengthen our quality assessment in the future, we propose to conduct objective assessments of various generics procured as available stock items. This approach will allow us to more closely and objectively monitor the quality of acquired drugs in subsequent rounds.

Based on the success of our piloting of pooled procurement in the network, conducting such negotiations may be relevant at a larger scale for oncology drugs, such as through the national health authority, as that will enhance the bargaining power as well as have far-reaching impact on access and affordability across the entire national network. Negotiation on a national level could also address the challenges of vendor monopoly or patented drugs supplied by a single vendor. Furthermore, to determine the final price for innovator and single vendor drugs, a comprehensive evaluation of the available literature on efficacy and safety data is crucial. If a drug meets the threshold for significant clinical benefits, cost-effectiveness assessment using adaptive health technology can provide guidance for negotiating prices.

We also plan to expand the second cycle of pooled procurement to include consumables, equipment and electronic health record systems. While these systems will require a slightly different process to evaluate quality and demand, and to gain group consensus on specifications and requirements, they have the potential to positively disrupt high costs of cancer care across India. We are interested to see if the price discovery cell model, when applied in group negotiations in other low- and middle-income countries, will yield similar price reductions and quality improvement, to facilitate a shift towards pooled procurement as the standard procurement method for all essential drugs, equipment and supplies in the future.
